# Pseudoaneurysm and Arteriovenous Fistula After Femoral Puncture During Atrial Fibrillation Ablation: An Access‐Site Pitfall in Large‐Bore Femoral Procedures

**DOI:** 10.1002/joa3.70384

**Published:** 2026-06-07

**Authors:** Hikaru Masuda, Ryuta Watanabe, Yuya Koike, Masashi Tanaka, Yasuo Okumura

**Affiliations:** ^1^ Division of Cardiology, Department of Medicine Nihon University School of Medicine Tokyo Japan; ^2^ Department of Cardiovascular Surgery Nihon University School of Medicine Tokyo Japan

**Keywords:** arteriovenous fistula, atrial fibrillation, femoral artery puncture, pseudoaneurysm, pulsed field ablation

## Abstract

High femoral puncture during large‐bore femoral procedures can injure the inferior epigastric artery, causing pseudoaneurysm and arteriovenous fistula. This case highlights the need for careful access‐site selection using combined ultrasound and fluoroscopic guidance, even during pulsed field ablation.

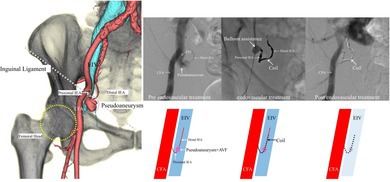

Pulmonary vein (PV) isolation with pulsed field ablation (PFA) for atrial fibrillation (AF) is an emerging therapy with myocardial selectivity that provides efficacy comparable to radiofrequency ablation while reducing complications [1]. However, vascular complications may still occur despite ultrasound‐ and fluoroscopy‐guided puncture, because multiple punctures and large‐bore sheaths are often required. We report an asymptomatic patient who developed both a pseudoaneurysm and an arteriovenous fistula (AVF) at a single femoral puncture site.

A 78‐year‐old woman with no past medical history underwent pulsed field ablation for symptomatic paroxysmal atrial fibrillation. Preoperative blood tests, transthoracic echocardiography, and three‐dimensional cardiac computed tomography (CT) revealed no abnormalities. She was 143.3 cm tall and weighed 50.7 kg, with a body mass index of 24.7 kg/m^2^, within the normal range for an Asian woman. Oral anticoagulation with edoxaban 30 mg/day was continued until the procedure, and PFA with a pentaspline catheter (FARAWAVE, Boston Scientific) was performed. Vascular access was obtained via the right internal jugular vein (7 Fr); two femoral venous sheaths, including an 8.5 Fr transseptal sheath (Swartz, Abbott, Abbott Park) and a 10 Fr sheath; and a 4 Fr sheath was placed in the right common femoral artery (CFA). Femoral access was obtained under ultrasound guidance, with fluoroscopic visualization of the femoral head used as an anatomical landmark. After transseptal puncture and left atrial pre‐mapping, the Swartz sheath was exchanged for a 16.8 Fr PFA sheath (FARADRIVE, Boston Scientific) with an over‐the‐wire technique. The activated clotting time was maintained between 300 and 350 s, with intravenous heparin. PV isolation with PFA was performed according to the standard protocol. For left atrial post‐mapping, the 10 Fr femoral venous sheath was exchanged for an 8.5 Fr transseptal sheath, to confirm four‐PV isolation. Hemostasis was achieved with a Z‐suture and 15 min of manual compression. After confirming no shunt bruit, compression hemostasis was performed with 8 h of strict bed rest, followed by position changes allowed, for a total of 15 h. On postoperative day 1 (POD1), after discontinuation of compression, there was no rebleeding, puncture site pain, hematoma or subcutaneous hemorrhage. However, auscultation revealed a continuous murmur with a diastolic component. Ultrasonography revealed a shunt flow suspicious for AVF, and contrast‐enhanced CT was performed. Contrast‐enhanced CT revealed a pseudoaneurysm of the right inferior epigastric artery (IEA) and an AVF with the adjacent external iliac vein (EIV) (Figure 1). Therefore, additional manual compression under ultrasound guidance and renewed compression hemostasis were performed until the following morning. Oral anticoagulation with DOAC was continued without interruption. On POD2, follow‐up CT showed no significant change, and endovascular treatment was performed (Figure 2). Under local anesthesia, an 8 Fr, 45 cm guiding sheath (Destination, Terumo) was inserted from the left CFA to the right external iliac artery, and a steerable microcatheter (Leonis 0‐0‐Mova, SB Kawasumi) was advanced into the right IEA. The distal IEA was embolized with four detachable coils (Target XL, 3 mm × 9 cm; Stryker) and one pushable coil (C‐Stopper, 60 mm; Piolax). The proximal IEA was then embolized using two Target XL coils (4 mm × 12 cm and 3 mm × 6 cm) with balloon assistance (Selecon MP, Terumo). Final angiography confirmed resolution of the pseudoaneurysm and AVF. On POD4, the patient remained asymptomatic, and no vascular bruit was detected; therefore, she was discharged home.

**FIGURE 1:**
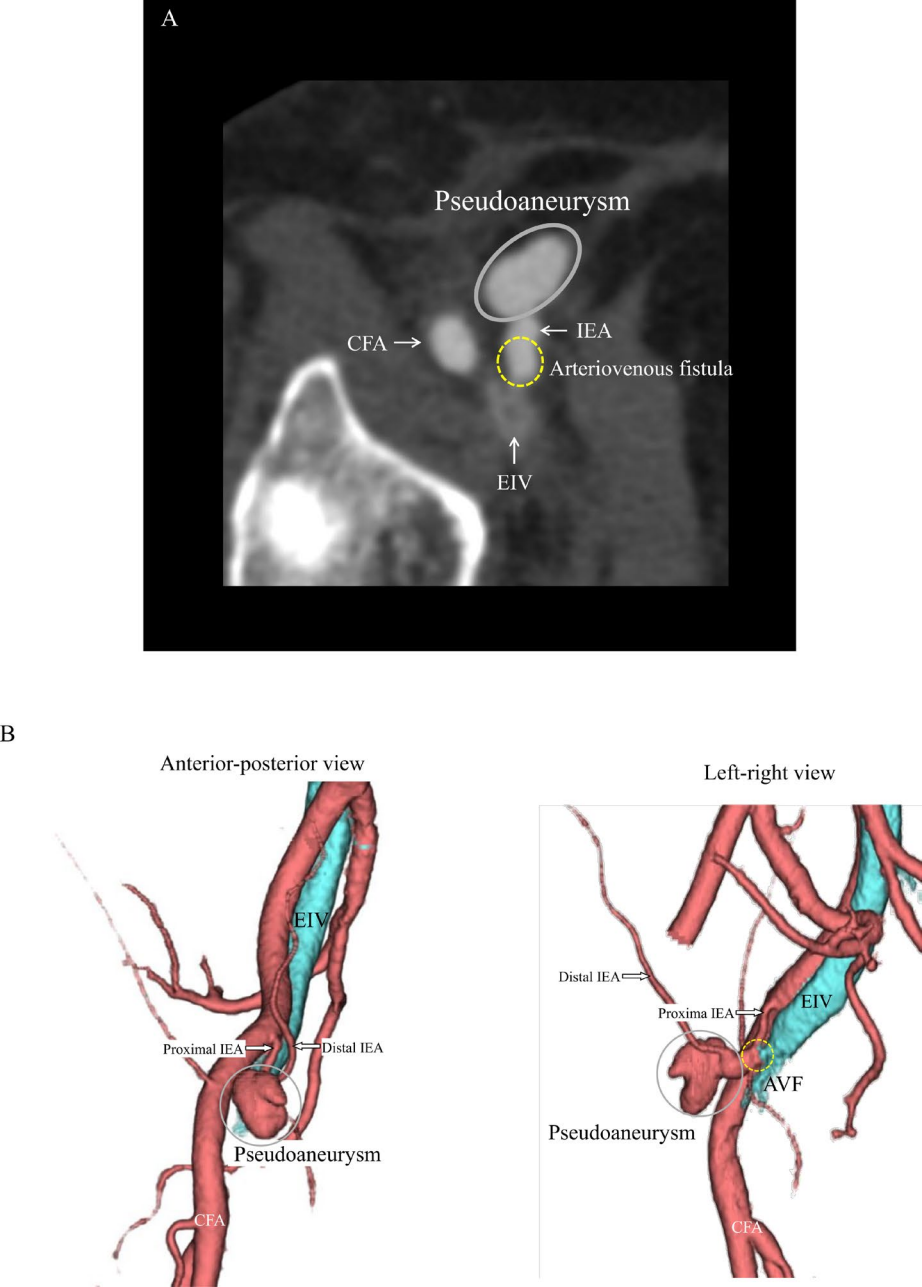
Horizontal plane (A) and three‐dimensional reconstruction (B) of computed tomography angiography. A pseudoaneurysm (gray circle) from the inferior epigastric artery and an accompanying arteriovenous fistula (yellow dashed circle) communicating with the adjacent vein were identified. AVF, Arteriovenous fistula; CFA, Common femoral artery; EIV, External iliac vein; IEA, Inferior epigastric artery.

**FIGURE 2:**
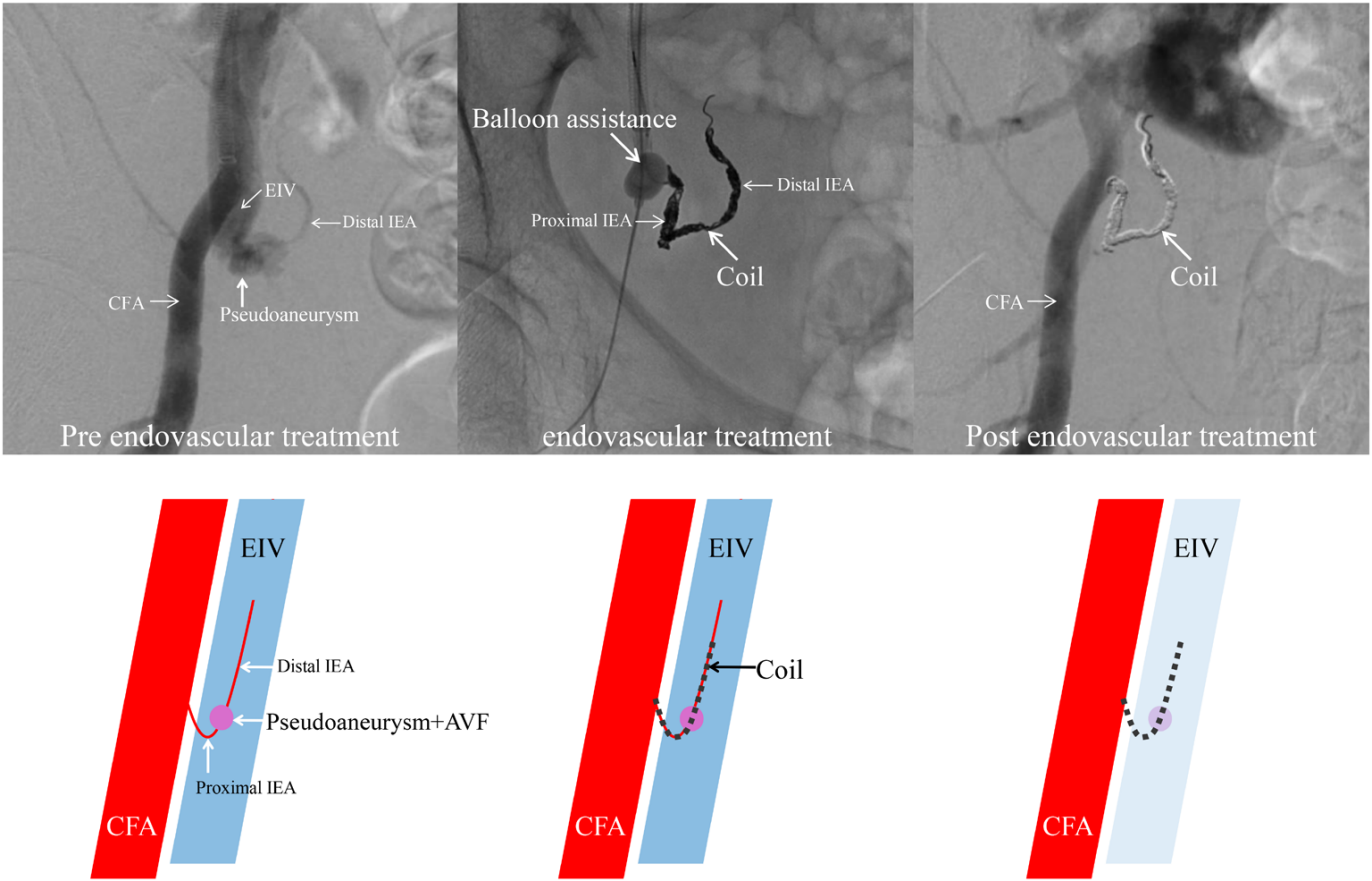
Fluoroscopic image and schema of endovascular treatment. Following coil embolization of the pseudoaneurysm and arteriovenous fistula originating from the inferior epigastric artery, postoperative angiography confirmed complete resolution of both lesions. AVF, Arteriovenous fistula; CFA, Common femoral artery; EIV, External iliac vein; IEA, Inferior epigastric artery.

Catheter ablation with PFA has a lower incidence of complications than conventional thermal ablation, owing to its nonthermal mechanism. However, vascular access complications (VACs) have been reported. Although this case occurred during PFA, the mechanism was related to high femoral puncture and large‐bore sheath manipulation rather than the energy source itself and is therefore relevant to other large‐bore procedures, including leadless pacemaker implantation, left atrial appendage closure, and structural interventions [2]. This case also underscores the importance of careful postprocedural auscultation, as VACs may remain clinically silent. The distinguishing feature of this case is the simultaneous occurrence of pseudoaneurysm and AVF in the IEA region, whereas its educational value lies in the preventive message for electrophysiologists. In addition, medial deviation of the CFA may increase the risk of IEA injury during femoral puncture.

Although ultrasound‐guided femoral access has been associated with lower VAC rates, a larger sheath diameter and multiple venous access sites remain important risk factors [3]. Although this complication was unrelated to the PFA energy source itself, it was likely associated with procedure‐related factors encountered during PFA [1]. In the present case, despite real‐time ultrasound guidance and fluoroscopic confirmation of the femoral head, vascular injury to the IEA during large‐bore sheath manipulation likely resulted in a pseudoaneurysm and an AVF. The AVF was identified as a communication between the injured arterial segment and the adjacent vein on imaging. Overall, this complication was probably multifactorial, involving patient‐related factors including advanced age, small body size, and continued anticoagulation, and procedure‐related factors including high puncture near the superior margin of the femoral head, multiple puncture sites, large‐bore sheath use up to 16.8 Fr, and sheath exchanges. DOAC was continued because thromboembolic risk after AF ablation was prioritized. The distal external iliac artery, the source of the IEA, runs along the superior margin of the femoral head; therefore, puncture near this margin should be avoided, particularly when the femoral artery is medially deviated, as in the present case (Figure 3) [4]. A previously reported ablation‐related IEA AVF resolved spontaneously, whereas the present case required coil embolization for an accompanying pseudoaneurysm, suggesting variable severity and management [5]. Recommended measures include anterior–posterior fluoroscopic confirmation of the femoral head with real‐time ultrasound identification of the CFA and adjacent femoral vein; when available, preprocedural CT may also help identify unfavorable arterial anatomy.

**FIGURE 3:**
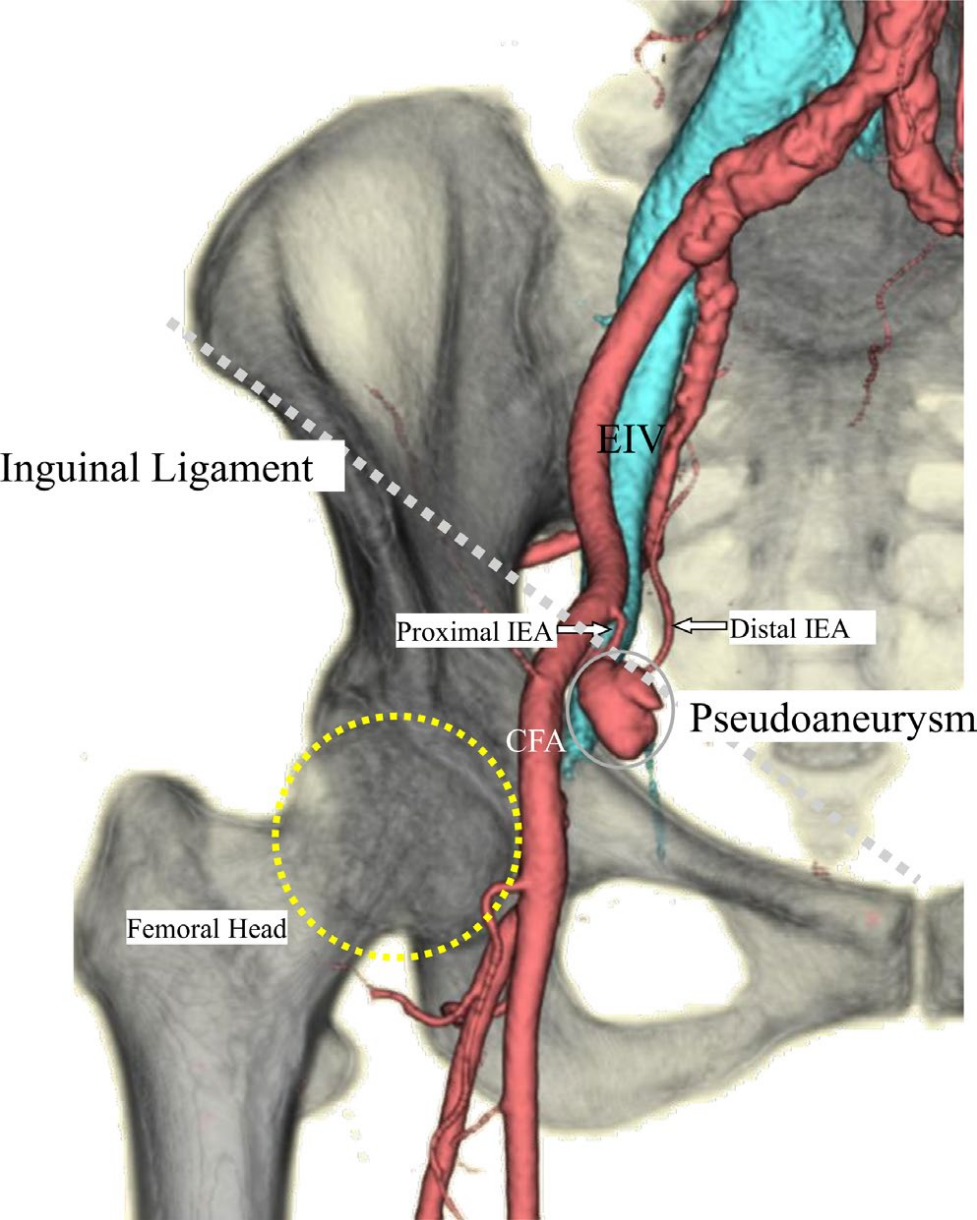
Anatomical relationship of the inguinal ligament, femoral head, and femoral artery system. At the superior margin of the femoral head (yellow dashed circle), the IEA descends toward the inguinal ligament (gray dashed line). In cases where the CFA deviates medially, the length of the IEA potentially at risk of vascular complications is longer. CFA, Common femoral artery; EIV, External iliac vein; IEA, Inferior epigastric artery.

High femoral puncture near the superior margin of the femoral head may injure the distal external iliac artery or the origin of the IEA, causing pseudoaneurysm and AVF. Combined real‐time ultrasound guidance and anterior–posterior fluoroscopic confirmation of the femoral head should be considered during femoral puncture, particularly when large‐bore sheaths are used.

## Disclosure

Dr. Yasuo Okumura has received research funding from Medtronic Japan, MicroPort CRM Japan, Bayer Healthcare, has accepted remuneration from AstraZeneca and Johnson & Johnson, and belongs to the endowed departments of Boston Scientific Japan, Abbott Medical Japan, Japan Lifeline, Medtronic Japan, and BIOTRONIK Japan. Other authors: None.

## Ethics Statement

The requirement for institutional review board approval was waived by the Institutional Review Board of Nihon University School of Medicine because this report describes a single de‐identified case.

## Consent

Written informed consent was obtained from the patient for publication of this report and accompanying images.

## Conflicts of Interest

The authors declare no conflicts of interest.

## Data Availability

The data that support the findings of this study are available from the corresponding author upon reasonable request.
